# Energy-saving method for technogenic waste processing

**DOI:** 10.1371/journal.pone.0187790

**Published:** 2017-12-27

**Authors:** Bayandy Dikhanbaev, Chandima Gomes, Aristan Bayandievich Dikhanbaev

**Affiliations:** 1 Department of Heat and Power Energy, Kazakh AgroTechnical University, Astana, Kazakhstan; 2 Department of Electrical and Electronics Engineering, Universiti Putra Malaysia, Serdang, Malaysia; The Education University of Hong Kong, HONG KONG

## Abstract

Dumps of a mining-metallurgical complex of post-Soviet Republics have accumulated a huge amount of technogenic waste products. Out of them, Kazakhstan alone has preserved about 20 billion tons. In the field of technogenic waste treatment, there is still no technical solution that leads it to be a profitable process. Recent global trends prompted scientists to focus on developing energy-saving and a highly efficient melting unit that can significantly reduce specific fuel consumption. This paper reports, the development of a new technological method—smelt layer of inversion phase. The introducing method is characterized by a combination of ideal stirring and ideal displacement regimes. Using the method of affine modelling, recalculation of pilot plant’s test results on industrial sample has been obtained. Experiments show that in comparison with bubbling and boiling layers of smelt, the degree of zinc recovery increases in the layer of inversion phase. That indicates the reduction of the possibility of new formation of zinc silicates and ferrites from recombined molecules of ZnO, SiO_2_, and Fe_2_O_3_. Calculations show that in industrial samples of the pilot plant, the consumption of natural gas has reduced approximately by two times in comparison with fuming-furnace. The specific fuel consumption has reduced by approximately four times in comparison with Waelz-kiln.

## Introduction

Dumps of metallurgical enterprises of Kazakhstan have accumulated about 20 billion tons of technogenic waste products of mining-metallurgical complex, over the years. Out of them, the wastes of non-ferrous metals make up to 10.1 billion tons and ferrous materials up to 8.7 billion tons. The concentration of valuable components in them is not much lower than that in most natural ores. Annually, about 700 million tonnes of industrial wastes are generated in Kazakhstan alone [1]. As the reserves of rich polymetallic ores in the country will be sufficient only for 25–30 years [2], there is a noticeable trend in the last decade in producing precious metals from recoverable resources, technogenic wastes and pari passu from unconventional raw material sources. With such scientific interest arising, the treatment of waste products with energy-saving and less complex processing modes has become a significant and important scientific frontier in extracting valuable materials, at present.

Sources of such technogenic waste sources are; impounded mill tailings of sulphide copper-nickel ores, old pyrrhotine concentrates (OPC), impounded magnetite concentrates, slag-dust dumps of the mining and metallurgical companies and technogenic platinum-metal chromite placer deposits of many parts of former Soviet Union territories [3, 4].

Several techniques of extracting valuable metals and minerals from both technogenic wastes and rich ores have been proposed during the last few decades. One of the popular technique of extracting valuable ore components is the leaching and sorption via processing of fine ore fractions where leaching, sorption-desorption and electrolysis parameters in cyanide processes play a key role in increasing the process efficiency [5].

Methods for concentrating precious and nonferrous metals in multicomponent samples of bottom deposits of slag product storage ponds have also been investigated in the recent past [6–8]. Methods of adhesive flotation using tributyl phosphate and straw oil, collective melting of sulfatization operation, absorption extraction of rare platinoids and preparation of rich platinum-metal concentrates, have been studied in this regard [6–8].

Chantries et al. [9] has discussed galvano-coagulation zinc recovery technology from mine and waste dump water. The studies included thermodynamic substantiation, revealing kinetic regularities of selective separation of metals, sediment phase composition and structure analyses, laboratory and laboratory-batch testing on synthetic brine and real water [9]. One could also find in the literature, the methods proposed for liquid-liquid extraction and purification of niobium, tantalum and titanium from process solutions of loparite, perovskite and sphene concentrate decomposition with sulphuric and hydrochloric acids; niobium from lithium niobate production wastes decomposed by hydrochloric acid; and tantalum from tantalum capacitor and heat-resistant alloy wastes [10]. Extraction of metals into a solution from compound mineral raw materials using electrochemical leaching is also a prominent method used by some scientists. In such cases, leaching reagent (such as sodium hyposulfite) has been obtained simultaneously with the reaction of resolving metals in a solution of sodium alkali. The source of sulphur for obtaining the leaching reagent is most often a composite sulphur graphite [11].

Despite the above studies on the subject, only a few types of research in the literature pay serious attention to the optimizing of energy sources required for the extraction process and minimizing the emission of undesired components to the atmosphere. In case, if the metal is produced from technogenic waste the specific consumption of primary fuel (suitably CO_2_ emissions) increases 2–3 times, in comparison with their production from rich natural raw material [12]. In the field of technogenic waste treatment, there is still no technical solution, profitable enough to conduct the process satisfactorily. Therefore, the development of an energy-saving method of waste product processing by the aid of highly efficient melting unit that reduces specific fuel consumption and emission of CO_2_ and other greenhouse gases should be a priority in the energy management engineering. This study has been done in this backdrop and provides a new technology for the technogenic waste processing to extract valuable metals.

## Methodology

For conducting the study, permits were obtained from the Committee of Ecology and Sanitary-Epidemiological Service of Republic Kazakhstan. Pilot plant studies did not involve endangered or protected species. The study was carried out on first author’s (Bayandy Dikhanbayev) private land.

Based on scientific methods of physical and thermal similarity, method of affine modeling and analysis of technical and economic characteristics of both traditional method—bubbling layer of smelt, (applied in high-temperature processing of mineral raw materials as fuming-furnace, furnace of Vanyukov, furnaces of Кaldo-type–TBRC, Ausmelt, Sirosmelt) and advanced method—boiling layer of smelt (straight flow-vortex smelting chamber, reactor of boiling layer of smelt), a new technological method—called smelt layer with inversion phase (LIPh) has been developed. In this regard, a pilot plant of melting unit of the new generation called reactor of inversion phase–rotary kiln (RIPh-RK) has been introduced and a series of experiments have been conducted to validate the efficiency of the proposed method. Based on the results of experiments on the pilot plant, the industrial sample has been recalculated by the method of affine modelling.

The excavated zinc-containing slag was used in the experiments. The chemical composition of the material is (by percentage):

ZnO(3.5–10); PbO(0.1–1.15); Cu(0.6–1.0); FeO(7–8); Fe_2_O_3_(2–3); Fe_3_O_4_(23–24); SiO_2_(27–28); CaO(13–14); Al_2_O_3_(7–9); S(0.4–0.5).

Zinc in the slag is integrated with hard-to-restore and complex compounds such as silicates (Zn_2_SiO_4_), ferrites (ZnFe_2_O_4_), aluminium spinel (ZnAl_2_O_4_), sphalerite (ZnS), etc.

## Results and discussion

The mass transfer process in bubbling layer of smelt is given by the Eq (1):

M=βFΔCτ (1)

where:

*M*—flow of substance mass (kg);
*β*—mass-transfer coefficient in the liquid phase (m/s)
*F*—total surface of mass transfer (m^2^)
ΔC—gradient of impurities concentration at boundary of phases i.e. driving force of process (kg/m^3^)
*τ*–dwelling time of bubbles (s)

Eq (1) shows that to force the mass transfer in bubbling layer, it is required to increase the reaction surface, the driving force of process, the residence time of gas bubbles in the layer and the mass-transfer coefficient in the liquid phase.

As *M* and *β* are determined by the results of the process, for forcing the mass transfer, it is only possible to vary the factors *ΔC* and *τ*. The influence of *F* can be disregarded, due to the invariable hydrodynamic condition in the furnace.

However, in practice, particularly when slag fumigation takes place, the forcing possibility of mass transfer (by blowing through the layer) by using the indicated factors has some limits conditioned by the internal physicochemical processes. An example is the industrial test on the enlarged height of periodical action-fuming furnace of Chimkent lead plant [13]: With the regime of the bubbling layer of smelt, within 10 days 7564 tons of liquid and 1317 tons of cold slag are reprocessed. The furnace charge was increased from 75 tons up to 90 tons, and the oxygen content in the blast was increased from 21% up to 26%. For the maintenance of constant intensity of the blast, the consumption of natural gas was increased from 4700 nm^3^/h up to 5900 nm^3^/h and the coefficient of air consumption was reduced from α = 0.76 to α = 0.6. In this case, the medium consistency of reduction gases at the exit from the combustion chamber of the furnace was increased from 18.2% up to 25%.

However, despite the increases of the residence time of gas bubbles in the layer, *τ*, and driving force of process Δ*C*, noticeable productivity increase in Zn, with respect to fuel consumption, was not observed.

Table 1 presents the values of Gibbs energy (Δ*G*) and equilibrium constants (*K*) of reactions of the formation and decomposition of silicate and ferrite of zinc and reductions of zinc oxide and metallic zinc oxidation at the temperature of 1400°C [14]. From this point, according to [15], the decomposition of Zn_2_SiO_4_ and ZnFe_2_O_4_ begins.

**Table 1:** Thermodynamic characteristics of reactions in the case of temp = 1400°C.

No	Reactions	ΔG, kJ	K
1	2ZnO + SiO_2_ = Zn_2_SiO_4_	–35.73	13.062
2	ZnO + Fe_2_O_3_ = ZnFe_2_O_4_	–32.37	10.256
3	Zn_2_SiO_4_ = 2ZnO +SiO_2_	35.73	0.077
4	ZnFe_2_O_4_ = ZnO + Fe_2_O_3_	32.37	0.097
5	ZnO + CO = Zn^g^+ CO_2_	–9.5	1.977
6	Zn^g^+Fe_2_O_3_ = ZnO+2FeO	– 45.795	26.9
7	Zn^g^+Fe_3_O_4_ = ZnO+3FeO	–1.809	1.139

According to Table 1, the composition probability of Zn_2_SiO_4_ and ZnFe_2_O_4_ from ZnO, SiO_2_, Fe_2_O_3_ is significantly higher than that in the decomposition reaction of silicate and ferrite of zinc in these components. The average value of the equilibrium constant of forward reactions 1 and 2 are higher by a factor of 100 than that in backward reactions 3 and 4. The same is observed in reaction 6, of which the equilibrium constant is 10 times higher than that in reaction 5. Therefore, a higher probability may be expected in the portion expending Zn^g^ on the generation of ZnO in the melt, and ZnO in the generation of silicates and ferrites. Therefore, it can be assumed that in the above-mentioned bubbling layer of smelt, with the increase of bath mass (by enlarging the height of furnace), output capacity change of zinc relative to fuel consumption has not been observed. This may be due to the residence time of bubbles in layer caused by the regrouping between recombined molecules of ZnO, SiO_2_, Fe_2_O_3_, zinc silicates and zinc ferrites (according to the reactions given in Table 1)

Thus, the results of the above-described experiment follow that for preventing the influence of reactions 1, 2, 6, 7; it is necessary to decrease the residence time of slag in the zone of processing (suitably the bath mass). For stimulation reactions 3, 4, 5; it is necessary to decrease the dwelling time of smelt and increase the temperature and reduction potential of gases in the treatment zone.

**Fig 1:**
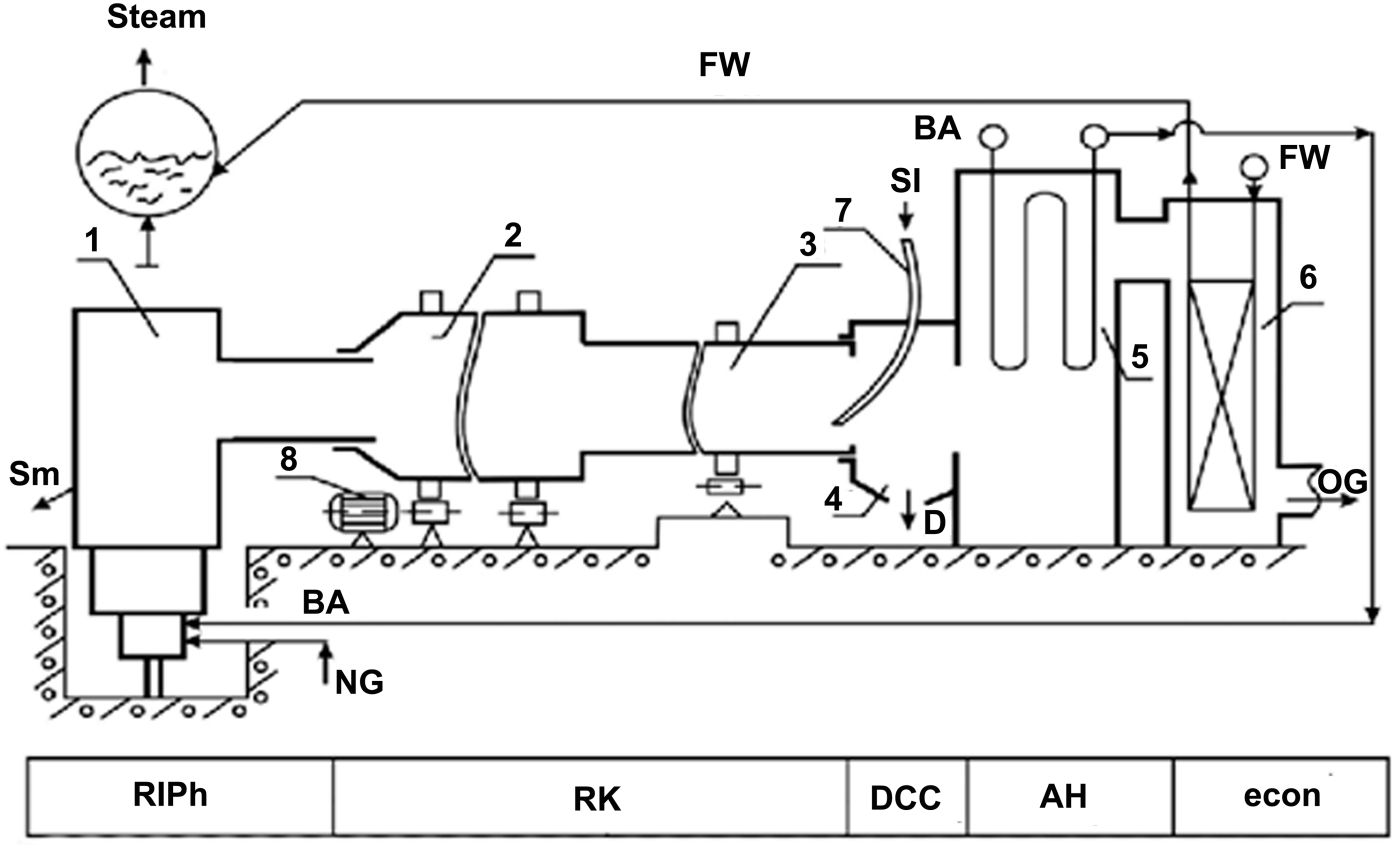
Basic circuitry of existing pilot plant. 1—reactor of inversion phase (RIPh), RK- rotary kiln, 2 –discharging and 3—loading parts of rotary kiln, 4 –dust collector chamber (DCC), 5—air heater (AH), 6—economizer (econ), 7 –slag loading tube, 8—drive of rotary kiln, Sl-slag, Sm-smelt, BA-blowing air, NG-natural gas, D-dust, FW-feed water, OG–off gases.

**Fig 2:**
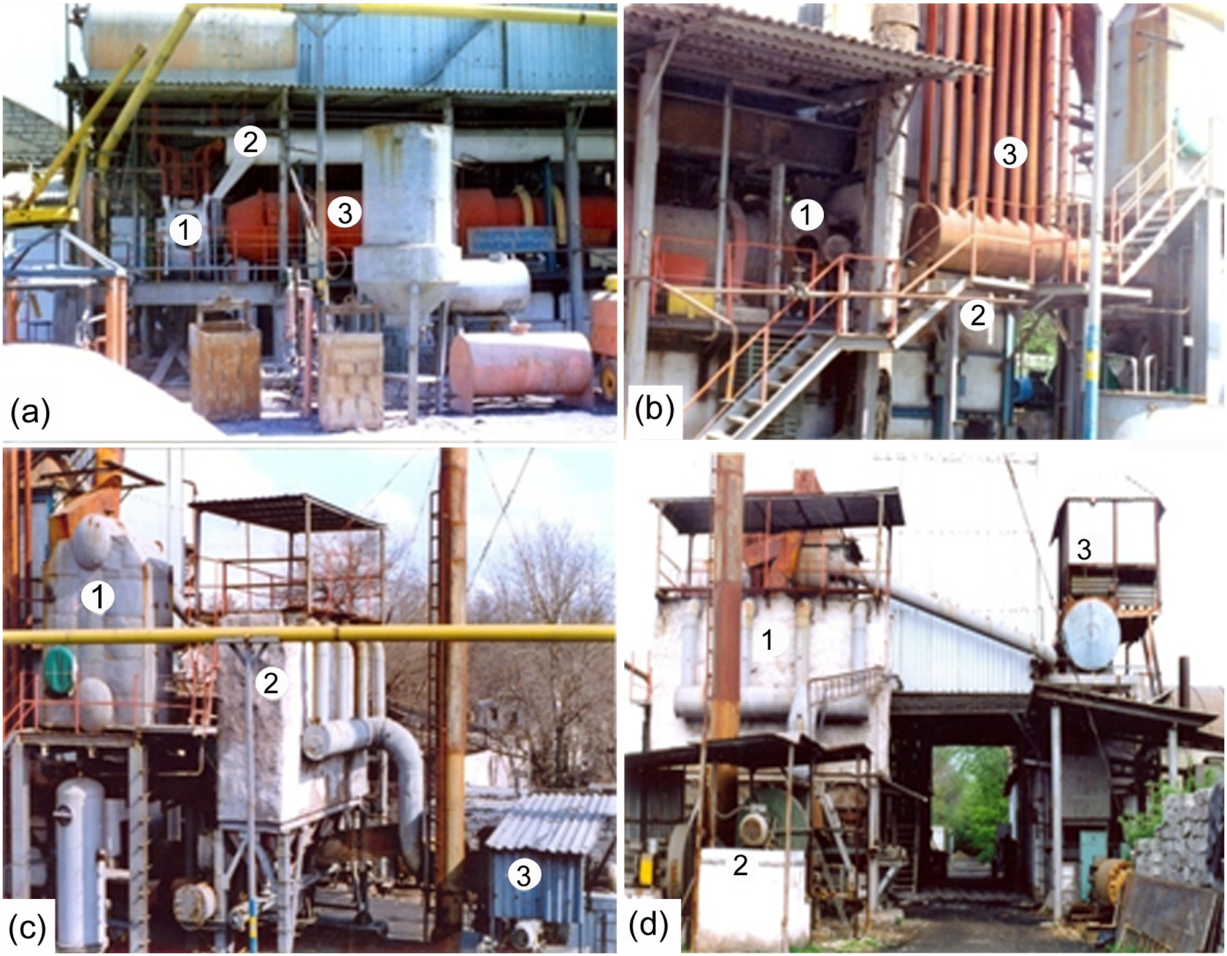
Main components of the existing installation. (a). reactor inversion phase (1), rotary kiln (2), by-pass (3); (b). dust collector (1), air heater (2), cooler (3); (c). economizer (1), bag filters (2), smoke suckers (3), (d). bag filters (1), smoke suckers (2), cooling tower for cooling of circulating water and a multi-nozzle grid of reactor inversion phase (3).

In the experiments conducted at the reactor of inversion phase of rotary kiln based pilot-plant (Fig 1), the main parameters of the new method of processing solid slag and the smelt layer with inversion phase by the developed boiling layer of melt are fundamentally different from the structure of the layer and achieving results by the traditional bubbling method, [16–18]. The main components of the plant are given in Fig 2.

**Fig 3:**
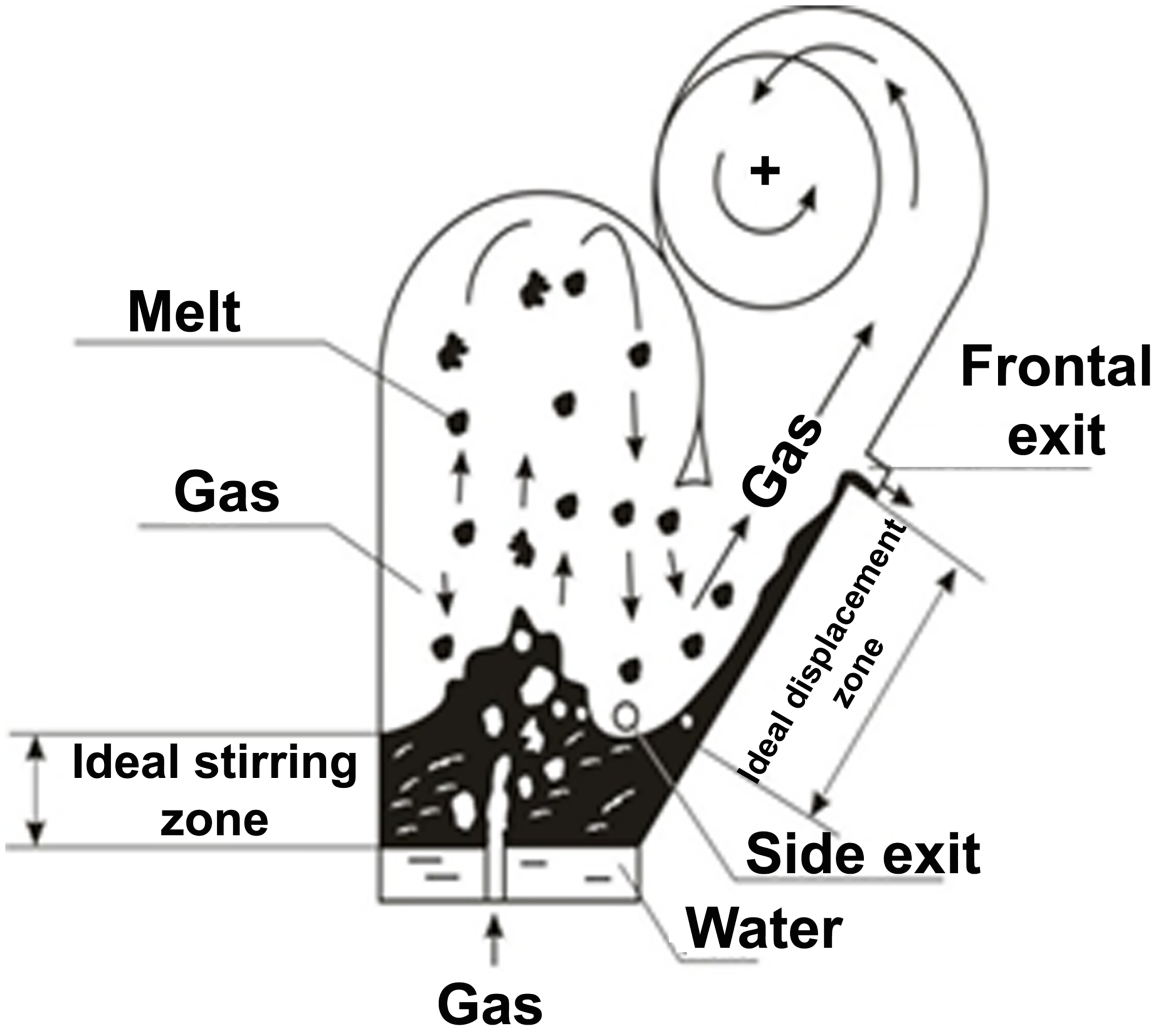
Principal design scheme of the reactor and physical picture of the inversion phase layer.

**Fig 4:**
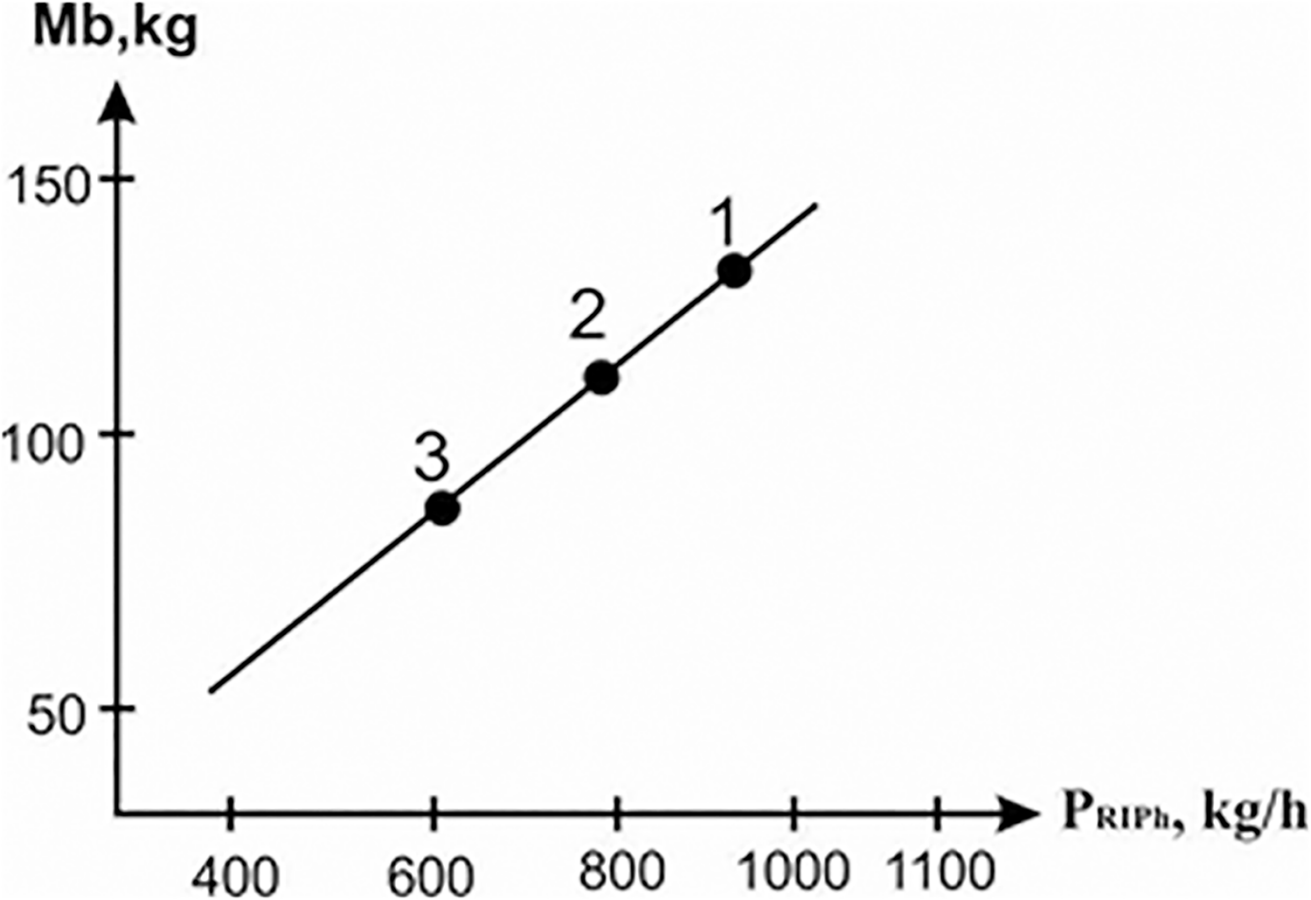
Experimental dependence of bath mass on the slag productivity of reactor inversion phase. 1. *I*_*noz*_/*G*_*b*_ = (0.26); 2. *I*_*noz*_/*G*_*b*_ = (0.42); 3. *I*_*noz*_/*G*_*b*_ = (0.44).

It is commonly known that the regime of boiling layer smelt by the analogy of the boiling fluidized solid particles is characterized by the condition known as gas–continuous/liquid–discrete. The constant localizing of the continuous gas phase and discrete liquid phase without any inversion is known as liquid–continuous/gas—discrete. The boiling layer of smelt (B_oil_SL), by definition, belongs to the regime of the ideal stirring. The inversion phase layer of smelt (LIPh) is formed when the jet of gas expires into the stationary layer of smelt with the formation of the gas-liquid emulsion, where the gas phase is in the liquid melt in the form of bubbles. Then when the gas is expanded into the free space, the liquid phase turns into discrete and the gas phase into the continuous condition. After that when the vertically down-take approach of the melt surface is reached, the liquid discrete phase becomes continuous again. The effectiveness of the zinc extraction in the inversion phase layer of smelt increases in the interaction zone of the gas-droplet flow output with the surface of the displaced melt, which raises up the oblique towards the tap hole (As shown in Fig 3). When the slag productivity is altered, in contrast to the bubbling and boiling smelt layers of which the bath mass is not dependent on the reactor’s slag productivity, in the smelt layer with the inversion phase, the bath mass changes linearly, which characterizes the inversion phase layer of the combination of two regimes. This can be described as the ideal stirring and ideal displacement (As shown in Figs 3 and 4).

In this paper, the following symbols and abbreviations have been used: *Zn*^*in*^, *Zn*^*fin*^–initial and final concentration of zinc, *W*_*noz*_/*W*_*pg*_−the ratio of the gas velocity in the nozzles of the purging grid in m/sec, to the gas velocity given to the area of he purging grid in m/sec; *I*_*noz*_/*G*_*b*_−the ratio of the gas impulse through the nozzles in kg∙m/sec^2^, to the weight of the molten bath on the grid in kg∙m/sec^2^; *M*_*в*_ −mass of the bath on the grid in kg; *α*_*com*_−coefficient of discharge of oxidant in the combustion chamber; h_out_−height of the outlet tap of the reactor; *Е*–extraction degree of zinc from the melt as a percentage (%); *τ*_*dw*_ −dwelling time of slag in the layer of inversion phase in minutes; *P*_*RIPh*_ −slag productivity of reactor inversion phase in kg/h; *В*_*ng*_ −reactor’s natural gas consumption in nm^3^/h; *V*_*a*_ −reactor’s blowing air consumption in nm^3^/h; $V_{O_{2}}$ –reactor’s oxygen consumption in nm^3^/h; *φ*–gas content in the layer; *t*_*a*_ −temperature of combustion air, °C; *t*_*s*_, *t*_*m*_ −temperature of slag after the rotary kiln and temperature of melt after reactor, °C; $t_{og}^{RIPh},t_{og}^{RK},t_{og}^{AH},t_{og}^{BF}$ –temperatures of outgoing gases after reactor, rotary kiln, air heater and bag filter, respectively, °C; lat.–lateral tap hole that is located on a vertical caisson of the shaft of the reactor; fr–frontal tap hole that is located on a sloping caisson of the separation chamber of the reactor; B_ub_SL–bubbling smelt layer; B_oil_SL–boiling smelt layer; LIPh–smelt layer with the inversion phase.

Work reported in [19] studied the regime of the boiling smelt layer, when processing the liquid slag from the electric settler of the lead furnace, in the range of change of similarity: *I*_*noz*_/*G*_*b*_ = 0.09–0.19; *W*_*noz*_/*W*_*pg*_ = 12.25. To process the solid dump slag in the unit reactor inversion phase of rotary kiln, a series of experiments were conducted to define the extraction level of zinc from slag when the smelt layer, according to the hydrodynamic characteristics, conforms bubbling smelt, boiling smelt layers and smelt layer with the inversion phase; *I*_*noz*_/*G*_*b*_ < 0.09, *W*_*noz*_/*W*_*pg*_ = 12−25, regime of bubbling smelt layer;

*I*_*noz*_/*G*_*b*_ = 0.09−0.19, *W*_*noz*_/*W*_*pg*_ = 12−25, regime of boiling smelt layer;

*I*_*noz*_/*G*_*b*_ > 0.09, *W*_*noz*_/*W*_*pg*_ = 12−25, the regime of smelt layer with inversion phase.

The averaged results of the experiments conducted within the interval *I*_*noz*_/*G*_*b*_ = (0.0577−0.26) and *W*_*noz*_/*W*_*pg*_ = 12−25 are given in Tables 2–4.

**Table 2:** Extraction degree of zinc in the regime of bubbling smelt layer (*I*_*noz*_/*G*_*b*_ = 0.0577) < 0.09.

*Zn*^*in*^ (%)	9.63	*φ*	0.55
*B*_*ng*_ (nm^3^/h)	280	*t*_*s*_ (°C)	970
*V*_*a*_ (nm^3^/h)	1470	*t*_*m*_ (°C)	1410
*t*_*a*_ (°C)	340	$t_{og}^{RIPh}$ (°C)	1470
$V_{O_{2}}$ (nm^3^/h)	110	$t_{og}^{RK}$ (°C)	532
*I*_*noz*_ */G*_*ϐ*_	0.0577	$t_{og}^{AH}$ (°C)	413
*W*_*noz*_ */W*_*pg*_	17.55	$t_{og}^{BF}$ (°C)	200
*α*_*com*_	0.78	*τ*_*dw*_ (min)	22.7
*P*_*RIPh*_ (kg/h)	1490	*Zn*^*fin*^ (%)	7.28
*M*_*в*_ (kg)	553	*E* (%)	29

**Table 3:** Extraction degree of zinc in the regime of boiling smelt layer 0.09 < (*I*_*noz*_/G_*ϐ*_ = 0.091) < 0.19.

*Zn*^*in*^ (%)	9.63	*φ*	0.66
*B*_*ng*_ (nm^3^/h)	300	*t*_*s*_ (°C)	948
*V*_*a*_ (nm^3^/h)	1590	*t*_*m*_ (°C)	1401
*t*_*a*_ (°C)	300	$t_{og}^{RIPh}$ (°C)	1467
$V_{O}{}_{{}_{2}}$ (nm^3^/h)	95	$t_{og}^{RK}$ (°C)	510
*I*_*noz*_ */G*_*ϐ*_	0.091	$t_{og}^{AH}$ (°C)	380
*W*_*noz*_ */W*_*pg*_	20.5	$t_{og}^{BF}$ (°C)	205
*α*_*com*_	0.75	*τ*_*dw*_ (min)	13.4
*P*_*RIPh*_ (kg/h)	1139	*Zn*^*fin*^ (%)	6.21
*M*_*в*_ (kg)	255	*E* (%)	40

**Table 4:** Extraction degree of zinc in the regime of smelt layer with inversion phase 0.19 < (*I_noz_*/*G_ϐ_* = 0.26).

*Zn*^*in*^ (%)	9.63	*φ*	0.75
*B*_*ng*_ (nm^3^/h)	280	*t*_*s*_ (°C)	960
*V*_*a*_ (nm^3^/h)	1512	*t*_*m*_ (°C)	1425
*t*_*a*_ (°C)	320	$t_{og}^{RIPh}$ (°C)	1480
$V_{O}{}_{{}_{2}}$ (nm^3^/h)	128	$t_{og}^{RK}$ (°C)	494
*I*_*noz*_ */G*_*ϐ*_	0.26	$t_{og}^{AH}$ (°C)	389
*W*_*noz*_ */W*_*pg*_	17.55	$t_{og}^{BF}$ (°C)	194
*α*_*com*_	0.83	*τ*_*dw*_ (min)	7.37
*P*_*RIPh*_ (kg/h)	1058	*Zn*^*fin*^ (%)	4.72
*M*_*в*_ (kg)	130	*E* (%)	55

From the data in Tables 2–4 we can see that the degree of restoration of zinc from the smelt increases with the change in the method of purging and is equal for:

bubbling smelt layer (*I*_*noz*_/*G*_*β*_ < 0.09),−*E* = 29%;

boiling phase (0.09 < *I*_*noz*_/*G*_*β*_ < 0.19)–*E* = 40%

inversion phase smelt layer (*I*_*noz*_/*G*_*β*_ > 0.19)–*E* = 55%

Shown in Tables 5 and 6 are the results of the experiments conducted in the range of changing the criteria *I*_*noz*_/*G*_*β*_ = 0.064–0.768, *W*_*noz*_/*W*_*pg*_ = 12 − 25.

**Table 5:** Results (Part-1) of the experiments.

No	Type of tap hole (type of regime)	*M*_*B*_ (kg)	P_RIPh_ (kg/h)	$\frac{I_{noz}}{G_{ϐ}}$	Zn^in^ (%)	Zn^fin^ (%)	*E* (%)	*B*_*ng*_ (nm^3^/h)
1	Lat. (B_ub_SL)	670	1200	0.064	4.3	3.27	29	288
2	Lat. (B_oil_SL)	461	1170	0.0896	9.63	7.12	31	280
3	Lat. (B_oil_SL)	180	573	0.196	9.63	6.63	36	300
4	Lat. (LIPh)	144	585	0.353	9.63	5.78	40	320
5	Fr. (LIPh)	75	636	0.56	9.63	3.66	67	300
6	Fr. (LIPh)	60	1000	0.578	10.96	4.49	64	270
7	Fr. (LIPh)	62	1200	0.768	10.96	4.18	62	316
8	Fr. (LIPh)	89	594	0.42	9.63	3.4	69	317
9	Fr. (B_oil_SL)	255	1139	0.091	9.63	5.75	40	310

**Table 6:** Results (Part-2) of the experiments.

No	V_a_ (nm^3^/h)	t_a_ (°C)	$V_{O_{2}}$ (nm^3^/h)	α_com_–	t_s_(°C)	t_m_,°C	$t_{og}^{RIPh}$ (°C)	τ_dw_
1	1786	305	57.6	0.789	920	1410	1465	33.5
2	1624	370	75	0.797	950	1400	1455	23.64
3	1590	320	126	0.8	945	1410	1465	21.05
4	2112	350	75	0.85	950	1415	1470	14.8
5	1590	340	107	0.773	910	1417	1475	7.07
6	1755	300	50	0.876	900	1405	1465	3.6
7	2038	350	75	0.833	960	1420	1470	3.1
8	2054	325	72	0.834	970	1405	1465	3.05
9	1800	340	80	0.78	970	1410	1470	13.4

According to Tables 5 and 6, points 2 and 3 in the conditions of boiling smelt layer discharge from the vertical side of the lateral caisson with the decrease of output capacity of layer on slag with 1170 kg/h up to 573 kg/h, it is possible to increase zinc recovery efficiency *E*, which does not typically exceed 36%.

To answer the question, “whether the zinc recovery efficiency increases under the conditions of the discharge from the vertical side of the caisson (lateral) during the regime of the inversion phase layer *I*_*noz*_/*G*_*b*_ = 0.19 −1.0″, a series of experiments were carried out. The results of these experiments are depicted in Tables 5 and 6 point 4. The test results show that the process organization during the mode of inversion phase layer, under the conditions of the discharge from the vertical caisson, does not increase the zinc recovery efficiency more than 40%.

To study the issue, “whether the zinc recovery efficiency increases under the conditions of the discharge from the leaning caisson (frontal) during the mode of boiling smelt layer”, a set of experiments were planned and executed and the parameters of one of them are depicted in Tables 5 and 6, point 9. As in conditions of the melt discharge from the leaning caisson, the mass of the bath was closely connected with the slag productivity of the reactor according to a leaner relation. The melt discharge from the frontal slag tap hole was regulated by hand to keep the *M*_*в*_ value satisfactory under the regime B_oil_SL (boiling smelt layer). The test results show that it is impossible to increase the zinc recovery efficiency more than 40% under the conditions of the discharge from the leaning caisson during the regime of boiling smelt layer—*I*_*noz*_/*G*_*b*_ = 0.09 −0.19. And only when the process operates in the regime of the inversion phase layer in the conditions of melt discharge from the leaning side of caisson (Tables 5 and 6, point 8) with the same level of productivity as in the regime of boiling melt layer—B_oil_SL (Tables 5 and 6, point 3), the zinc recovery efficiency increases up to 69%.

To choose the section of the leaning caisson where the zinc recovery efficiency increases, several experiments were conducted for different heights of the frontal slag tap hole in meters (0.7; 0.9; 1.1; 1.25). The regime parameters of the tests in Table 7.

**Table 7:** The results of testing for the slag with Zn^in^ = 9.63%.

h_out_ (m)	P_RIPh_(kg/h)	M_B_ (kg)	$\frac{I_{noz}}{G_{ϐ}}$	E (%)
0.7	1240	63	0.765	62
0.9	1200	62	0.768	62
1.1	1000	60	0.578	64
1.25	636	75	0.568	67

The results of the experiments shown in Table 7 depict that during the regime of inversion phase layer, the zinc recovery efficiency increases in the coverage area of the vertical falling of the gas flow drop on smelt film surface, rising along the lean in a direction to the slag tap. For this experiment, the coverage area is located in the range of slag tap with h_out_ = 0–0.7_M_. The height of the slag tap (under the conditions of the leaning- frontal caisson) out of the zone of interaction (gas flow drop of the rising smelt film) doesn’t influence the zinc recovery efficiency. This is the reason that at the slag tap with h_out_ = 0.7; 0.9; 1.1; 1.25 m, the rate of zinc recovery during the experiment remains approximately constant. A small increase of *E* with h_out_ in the range 1.1 m -1.25 m was detected with the decrease of the slag productivity of the reactor.

As shown in Table 5 the switch of the regimes from the bubbling, boiling layers of smelt to the inversion phase smelt layer along with the growth of blast intensity of layer (*I*_*noz*_/*G*_*β*_) shortens the duration of slag stay in the layer τ_dw_ and increase the zinc recovery efficiency *E*.

The latter fact can be explained by the following physicochemical factors (Tables 2–4). The duration of slag staying in the smelt layer with the inversion phase, LIPh, (where τ = 7.37 min) is less than that in the boiling layer of smelt, B_oil_SL, (where τ = 13.4min) and bubbling layer of smelt, B_ub_SL, (where τ = 22.7 min). That allows the zinc vapour to leave the layer quicker than that to be oxidized in the melt by Fe_2_O_3_ and Fe_3_O_4_. Gas content in the LIPh (φ = 0.75) is more than that in the B_oil_SL (φ = 0.66) and BubSL (φ = 0.55). Thus, the probability of collision between the mixture of melt droplets and the renewed surface, producing zinc silicates and zinc ferrites from recombined molecules ZnO, SiO_2_, Fe_2_O_3_ in LIPh is less than that in the B_oil_SL. The zinc recovery efficiency increases in the LIPh because of the lack of mixture in the zone of ideal displacement during the process of interaction of the gas flow drop in the melt film.

The outcome of this study shows that, in the processing of complex on phase composition of excavated zinc-containing slag, the comparison of three technological principles in identical conditions (P_RIPh_ = const, I_noz_ = const) depicts the following. The efficiency of slag reduction sequentially increases in the regimes: the bubbling layer of smelt, the boiling layer of smelt and the smelt layer with inversion phase. An assumption has been made that because of the lesser value of the duration of slag stay, a relatively high value of gas content in the layer of inversion phase (LIPh) is found in comparison with B_ub_SL and B_oil_SL. Thus, in the recovering part of zinc in the zone of ideal displacement, the possibility of regeneration of zinc silicates and zinc ferrites from the recombined molecules ZnO, SiO_2_, Fe_2_O_3_ decreases.

When developing high-temperature processes and equipment based on new technological ideas, the researcher has to answer the following questions: how could the "cold model" data, calculated on the basis of a number of assumptions, be transferred into the pilot plant and how should the results of complex and labor-intensive tests of pilot plant be transferred into the industrial sample without having an excessive risk.

The method of similar physical analogues that requires a mathematical description of the studied processes and its subsequent analyses by using similarity theory is used in practice for determining the basic parameters of the pilot plant; thereby answering the first question.

However, while satisfying the most stringent stipulations of the modelling process, this method leaves one or more unanswered similarity requirements. Therefore, a strictly similar implementation of the industrial equipment based upon pilot plant testing is very approximate, albeit necessary.

To answer the second question, Klyuchnikov [20] proposed the method of affine physical models. According to Klyuchnikov [20], this method, just as the method of similar models, requires recalculation of the model's evaluation data for parity of uniform similarity criteria to the sample that is similar to the given model. However, this sample, denoted as provisory, together with the model, belongs to the affine series related to the forecasted sample and may have uncorrelated calculated parameters. Therefore, the affine model method requires calculated correction of the provisory sample to align its individual parameters with the proposed operating conditions of the sample under consideration.

For an example, it is necessary to re-calculate dimensions of the purge grid of melting reactor (the model) of output capacity *Q*_*mod*_ = 2 *t*/*h* on slag to its industrial sample with *Q*_*ind*_ = 12 *t*/*h*. Grid performances of the model in active purging zone: the pressure of blast p = 0.7 kg/sm^2^, number of nozzles n = 6, the diameter of a nozzle d = 45mm and distance between nozzles S = 150 mm. Under the term of hydrodynamic analogy; *W*_*noz*_/*W*_*pg*_ = *idem*, *I*_*noz*_/*G*_*b*_ = *idem*, the industrial sample needs n = 40, d = 45 and S = 150.

However, the nozzles with such dimensions could not be placed in active zone purging grid of industrial sample. Therefore, for the industrial sample, under condition of *W*_*noz*_/*W*_*pg*_ = *idem* was chosen as n = 60, d = 29 and S = 100.

In this case, the resistance of purging grid will enhance and consequently, the bath mass will be increased where *I*_*noz*_/*G*_*b*_ ≠ *idem*. After performing an additional experiment on the gas-liquid model of the provisory sample, the desired pressure of blast p = 1 kg/sm^2^, in compliance with the condition of *I*_*noz*_/*G*_*b*_ = *idem*, has been determined.

After broadening and refining the totality of considered factors in the mathematical description of processes [21] that occur in the reactor of the pilot plant and having performed the appropriate conversions of the simultaneous equations and the boundary conditions, we obtain the list of main similarity criteria for the thermal operation reactor of inversion phase (RIPh).

When the thermal operation of RIPh (the model) is similar to the industrial sample RIPh, they will show the following similarity criteria developed in the result of tests:

**1. Geometric similarity**

$$H/h_{0}=3.27;D_{c}/d_{out}=1.6.$$ (2)

**2. Hydrodynamic similarity.**

$$W_{noz}/W_{pg}=17.55;I_{noz}/G_{ϐ}=0.83$$ (3)

$$Ho=\tau_{mix}∙g/W_{noz}=0.3656$$

where Ho—homochronicity criterion (dimensionless mixing time)

**3. Similarity in specific productivity**

$$\frac{P_{V}^{\prime }.q_{use}.V^{LIPh}}{B(D+z)c_{og}t_{og}}=0.4243,\frac{P_{V}^{\prime \prime }.q_{use}.V^{LIPh}}{B(D+z)c_{og}t_{og}}=0.666,$$ (4)

Where $P_{V}^{\prime }=6050Kg/m^{3}∙h,$ specific productivity for E = 85%; $P_{V}^{\prime \prime }=9500Kg/m^{3}∙h,$ E = 65% for extracting zinc from the melt.

**4. Heat loss similarity.**

$$\frac{q_{loss}.F_{hot}}{B(D+z)c_{og\;}t_{og}}=0.76$$ (5)

Here, H—expanded height of layer, h_0_ –“calm” height of layer, *D*_*c*_/*d*_*out*_−diameters of the cyclonic section and the gas outlet port of the reactor, *p*_*v*_—specific productivity of reactor inversion phase, *q*_*use*_—productively used heat energy in reactor, *V*^*LIPh*^—volume occupied by inversion phase layer, *C*_*og*_, *t*_*og*_—specific heat and temperature of exhaust gases, *q*_*loss*_ −specific heat loss across the reactor lining, *F*_*hot*_ −reactor’s hot surface, *B*—reactor’s natural gas consumption, D–number reactor’s off gases per 1m^3^ of natural gas, *z-* amount of Zn^g^ taking part in reduction per 1m^3^ of natural gas (or per 1mole of natural gas).

Based on the evaluated similarity conditions between the model and the sample, we can calculate the sample parameters in the following sequence.

Setting an arbitrary value "B" for natural gas consumption in the process, we can determine the composition of exhaust gases by using formula [22]:

$$\begin{array}{l} \left(K-1\right)x^{2}+\left[K\left(B_{c}+C_{H_{2}}-2E_{O_{2}}-Z\right)+2E_{O_{2}}+Z\right]\chi -B_{c}\left(2E_{O_{2}}-B_{c}+Z\right)=0,\\ Z=\frac{P_{s}\Delta C_{z}\cdot 22.4}{81\cdot B},y=B_{c}–x\\ W=2E_{O_{2}}-B_{c}-\chi -Z,q=C_{H_{2}}-w,D=A_{N_{2}}+B_{c}+C_{H_{2}},\\ Zn^{g}=\frac{z}{D+z},CO=\frac{y}{D+z},\;H_{2}=\frac{q}{D+z} \end{array}$$ (6)

Here, K—equilibrium constant of reaction *CO*_2_ + *H*_2_ ⟷ *CO* + *H*_2_ *O*, *x*, *y*, *w*, *q*—number of moles of CO_2_, CO, H_2_O and H_2_, respectively, per 1 mole of natural gas. $A_{N_{2}},B_{c},C_{H_{2}},E_{O_{2}}$—respective number of moles of nitrogen, carbon, hydrogen and oxygen that took part in the process, per 1 mole of natural gas, CO, H_2_, Zng—absolute shares of these components in the exhaust gas.

Consumption of the unknown natural gas for the "industrial sample" ca n be determined by using the formula derived from the reactor′s thermal balance equation;

$$Β=\frac{Ps\left[C_{s}(t_{m}-t_{s})+q_{m}+q_{end}-\Delta C_{z}c_{s}t_{s}\right]+F_{hot}∙q_{loss}}{Q_{NG}+\alpha \;{Ʋ}_{a}^{o}\;c_{a}\;t_{a}-(D+z)c_{og}t_{og}+CO∙q_{co}+H_{2}q_{H_{2}}+{Zn}^{g}∙\;q_{Zn}}$$ (7)

Here, Ps–industrial reactor’s slag productivity, t_m_, t_s_ t_a_, t_og_—temperatures of molten slag, feed slag, blowing air and off gases, respectively, c_s_, c_a_, cog—specific capacity of feed slag, blowing air and off gases, respectively, *q*_*m*_—specific heat of slag melting, *q*_*end*_—specific endothermic effect of ZnO reduction, ΔCz−share of ZnO reduction, q_co_, q_H2_, q_Zn_g–specific heat of CO, H_2_ and Zn^g^–respectively, Q_NG_−natural gas heat capacity, α–blowing air consumption coefficient, v_a_^0^—theoretical necessary combustion air expense.

Let’s identify:

$$C_{s}(t_{m}-t_{s})+q_{m}+q_{end}-\Delta C_{z}\;c_{s}t_{s}]=a,$$

$$Q_{NG}+\alpha {Ʋ}_{a}^{o}∙c_{a}t_{a}-(D+z)c_{og}t_{og}+CO∙q_{co}+H_{2}q_{H_{2}}+{Zn}^{g}∙q_{zn}=ϐ,$$

then

$$B=\frac{Ps∙a+F_{hot}∙q_{loss}\;}{ϐ},$$ (8)

The empirical expression for the reactor′s hot surface, [16]:

$$F_{hot}=12.5∙H\sqrt{F_{pg}}+15∙F_{pg}$$ (9)

here, H–expanded the height of inversion phase layer, F_pg_−area of purge grid.

$$F_{pg}=\frac{B(D+z)\beta_{g}}{3600\;W_{pg}}$$ (10)

where *β*_*g*—_temperature coefficient of gas expansion inside the inversion phase layer.

Transforming Eq (9):

$$\frac{(D+z)\beta_{g}}{3600}=c,F_{pg}=\frac{B∙c}{W_{pg}}$$ (11)

Solving Eqs (8), (9), and (11) simultaneously, we derive the equation for fuel consumption:

$$(ϐ-15∙c∙q_{loss}∙W_{pg}^{-1})B-(12.5∙c^{0.5}∙q_{loss}W_{pg}^{-0.5})H∙B^{0.5}-{aP}_{s}=0$$ (12)

By varying values of H_j_ in (12), we find a series of values for B_i_. By substituting B in (11), we determine *F*_*pgi*_. The derived value of fuel consumption must satisfy the condition

$${{(H_{i}∙F_{pgi}=V^{LIPh},(V}^{LIPh})}^{ind}=\frac{{{(P}_{s})}^{ind}}{{{(P}_{v})}^{mod}},$$ (13)

We now compare the derived value of “B” with the previously set value of "B". If B_i_≠B, we repeat the calculation to derive this equality.

Table 8 shows the application of pilot plant test data results to the industrial sample by using affine modelling method. A waste slag from Ust-Kamenogorsk lead-zinc plant was used in the experiments.

**Table 8:** Recalculated results of pilot plant’s test data on its industrial sample.

1	Reactor’s productivity in terms of slag, t/h	Natural gas consumption (nm^3^ /h)		Reactor’s specific natural gas consumption (nm^3^ /tZn)		Reactor’s specific reference fuel consumption (kg r.f. /tZn)		Reactor’s hot surface, (m^2^)
		E = 65%	E = 85%	E = 65%	E = 85%	E = 65%	E = 85%	
2	5,0	560	851	112	170	134	204	23
3	12.0	1062	1600	89	133	107	160	40.5
4	25.0	1800	2680	72	107	86	128	67.7

According to [19], when processing liquefied slag with E = 65–75% at the Shimkent fuming furnace, with slag productivity 75 t/cycle (25t/h), the specific consumption of natural gas was 200–230 nm^3^/tZn.

According to [23], when processing granulated slag with E = 75–80% at the Waelz-kiln of Leninogorsk polymetallic plant, with slag productivity 31t/h, the specific consumption of reference fuel was 580–600 r.f./tZn.

Thus, in an industrial sample of the reactor inversion phase, the specific consumption of natural gas will be approximately 2 times lesser than that in the fuming furnace, and the specific fuel consumption is approximately 4 times lower than that in Waelz-kiln.

It should be noted that, in the pilot plant, water is used for two purposes, i.e. to cool the caissons of the reactor inversion phase, where the water is converted into a steam and to granulate the melt leaving the reactor. The water from the granulation pool is pumped into the cooling tower, and then it is reused to granulate the melt. Regulatory inspections do not detect excessive amounts of zinc in this recycled granulating water.

## Conclusions

Based on the analysis of technical and economic characteristics of both traditional method, (bubbling layer of smelt) and the advanced method (boiling layer of smelt), an entirely new technological method (called smelt layer with inversion phase, LIPh), was developed. Based on the proposed method, a pilot plant of melting unit of new generation termed “reactor of inversion phase if rotary kiln (RIPh-RK)” was constructed and a series of experiments have been carried out.

In the processing of excavated slag, by comparing three technological principles in identical conditions (P_RIPh_ = const, I_noz_ = const), it has been found that the efficiency of slag reduction increases sequentially in the regimes: the bubbling layer of smelt, the boiling layer of smelt and the smelt layer with inversion phase.

By using affine modelling method, the experimental data of pilot plant, for parity of uniform similarity criteria, were recalculated for parameters of an industrial sample.

The following conclusions could be drawn from the data of the outcome (Table 7) for the proposed unit when processing zinc-bearing slag:

1. Compared with the fuming furnace at Chimkent plant that processed liquefied slag, the specific consumption of natural gas will be cut down approximately by 50%.
2. Compared with the Waelz-kiln of Leninogorsk polymetallic plant that processed granulated slag, the specific consumption of reference fuel will decrease approximately by 4 times.
3. As the productivity of the unit in terms of slag will increase in the 5–25 ton/h range, the specific consumption of natural gas in that range will decrease approximately 1.5 times.

The details of the pilot plant and the raw data of the outcomes of the experiments could be referred in S1 Doc, S1 Video and S2 Doc respectively.

## Supporting information

S1 Doc Components and parameters of the pilot plant. (PDF)

S2 Doc The results of the experiments, with the release of the melt through the tap located on the inclined reactor caisson. (PDF)

S1 Video The operational activities of the pilot plant. (MP4)
