# Supplementary material for: Energy-saving method for technogenic waste processing
Source: PLoS One. 2017 Dec 27;12(12):e0187790. doi: 10.1371/journal.pone.0187790 (PMC5744926; doi:10.1371/journal.pone.0187790)
Supplement: S1 Doc — (PDF) [file pone.0187790.s001.pdf]

Components and parameters of the pilot plant

The study was carried out at author's (Bayandy Dikhanbayev) private land an area of 2300 m^2. The slag processing pilot plant comprised by following elements: reactor inversion phase (RIPh) - fire surface 10m^2; rotary kiln - length 9м, air heater (AH) heating surface – 200 m^2; blow - draught system including 2 turbo-blowers V=80m^3/min, P=1, 8bar, W=200кWt, each, 2 smoke suckers, W=45 кWt, each, bag filter, filtration surface – 200m^2; node of chemical water purification, natural gas and transformer station. The location of installation – Republic Kazakhstan, Shimkent city, Ismailov street ,16a, (refer S1 Video).

The phase inversion reactor consists of three elements: a melting chamber (Fig. 1), a nozzle grid (Fig. 2) and a combustion chamber for converting natural gas (Fig. 3). The insulating of the melting chamber is garnissagened, that is, self- lining by "freezing" the melt onto the walls of the chamber (photo 1).

In the combustion chamber there is incomplete combustion of natural gas to a temperature of 1900^{0}C, which is fed through the nozzle grid into the melting chamber at a speed of 500-600 m/s.

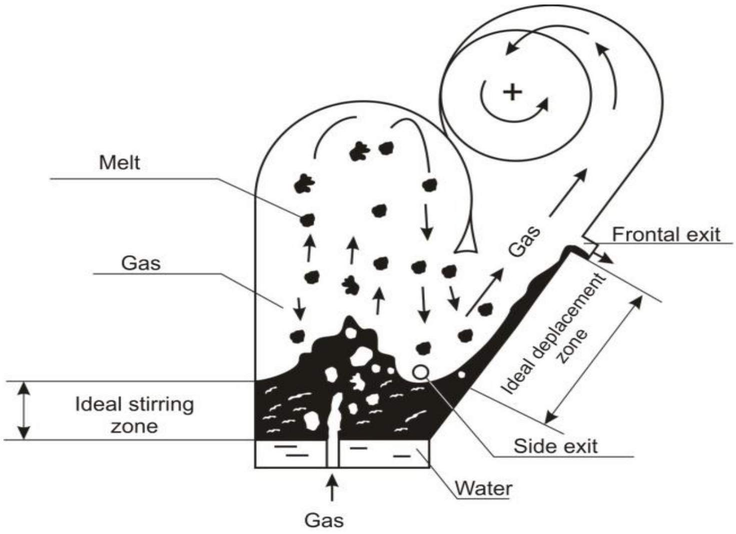

Fig 1 – **Principal design scheme of the reactor and physical picture of the inversion phase layer**

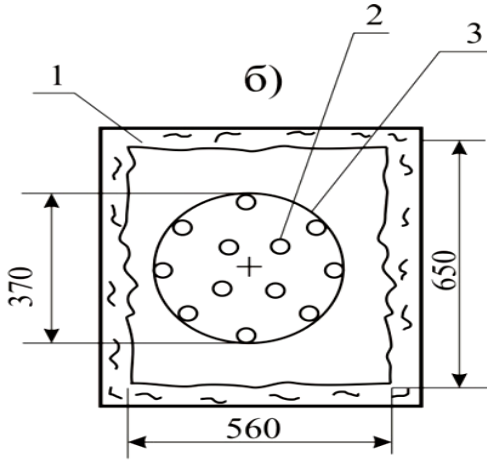

Figure 2 - **Nozzle grid of the reactor inversion phase.** 1 - garnissage lining, 2 - blowing grid nozzles, 3 - boundary of combustion chamber

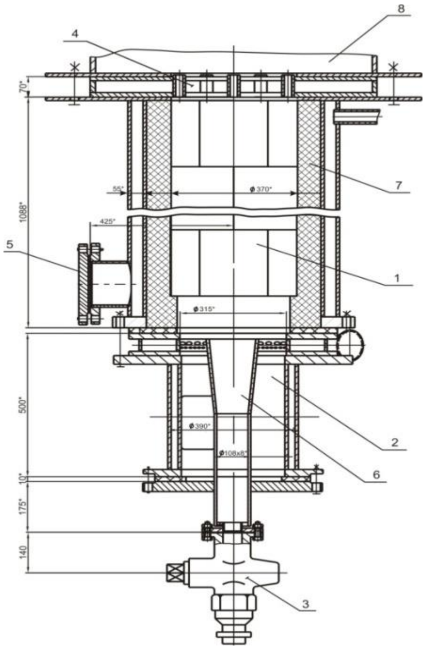

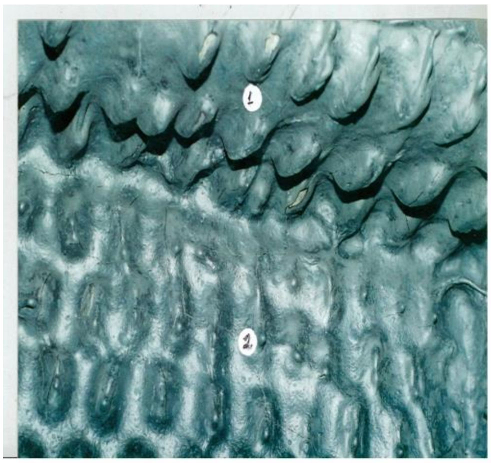

Photo 1 – **Garnissage lining of the reactor inversion phase**. 1 - ceiling caisson, 2 - vertical caisson.

Figure 3 - **Combustion chamber of reactor inversion phase** 1 - the combustion chamber, 2 - the mixing chamber, 3 - the pilot valve, 4 - the nozzle grid, 5 - the hatch for cleaning the water jacket of the chamber, 6 - the combustion stabilizer, 7 - the refractory bricks lining, 8- reactor inversion phase.

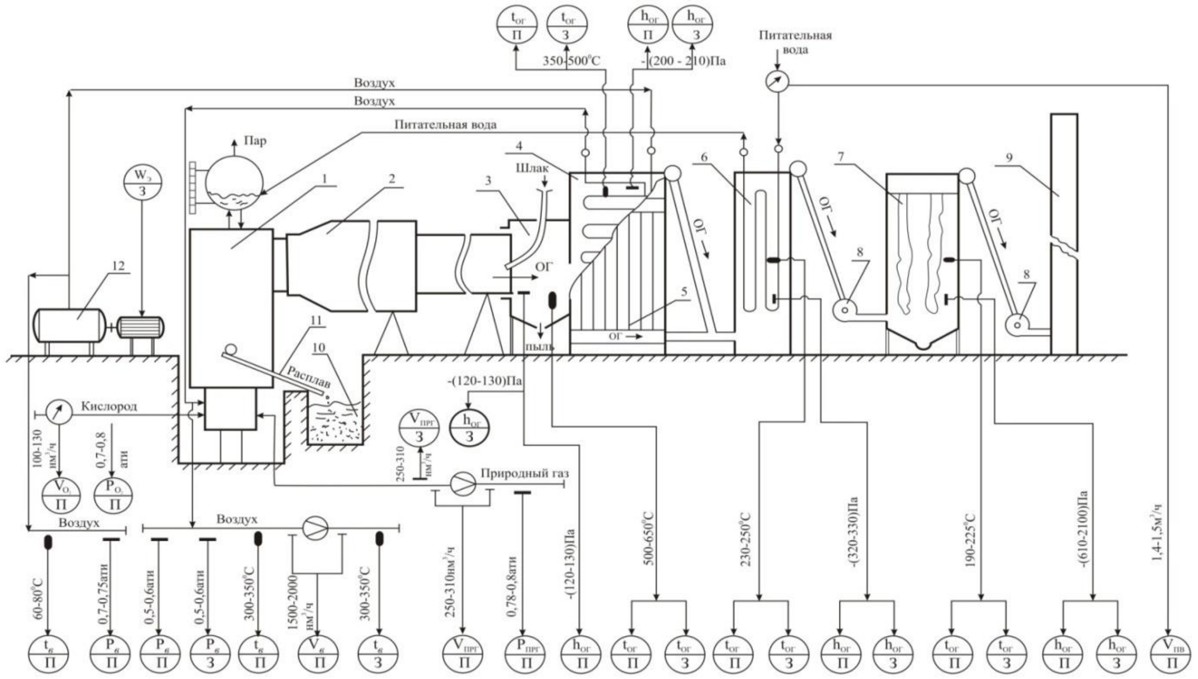

Figure 4 - **Scheme of measurements of the pilot plant**. 1 - reactor inversion phase, 2 - rotary kiln, 3 - dust chamber, 4 - air heater, 5 - cooler, 6 - feedwater heater for the reactor, 7 - bag filter, 8 - smoke exhausters, 9 - chimney, 10 - granulation pool, 11 - trough for a melt, 12 - blower.
