# Supplementary material for: Energy-saving method for technogenic waste processing
Source: PLoS One. 2017 Dec 27;12(12):e0187790. doi: 10.1371/journal.pone.0187790 (PMC5744926; doi:10.1371/journal.pone.0187790)
Supplement: S2 Doc — (PDF) [file pone.0187790.s002.pdf]

The results of the experiments, with the release of the melt through the tap located on the inclined reactor caisson

The experiments were carried out to determine the dependence of mass of bath on the slag productivity of the reactor in case of melt discharge from the inclined caisson. The results of the experiments show (see Fig. 1) that when the melt is discharged from the inclined part of the reactor, the dependence of mass of bath on the reactor's slag productivity varies on a straight linear law. As a result of the carried-out experiments in the regime of inversion phase layer with the melt release from inclined caisson, the degree of zinc extraction increased above 70% (see Table 1, experiments № 2 and № 3).

Conventional notations: P_{RIPh} - slag capacity of the reactor, M_B - mass bath of the reactor, B_{ng}- consumption of natural gas; I_C/G_B – the ratio of the gas impulse through the nozzles, to the weight of the molten bath on the grid; P_a - blown air pressure, τ_{dw} – dwelling time of slag in the reactor; E - zinc restoration degree, P_V - reactor's specific volume capacity.

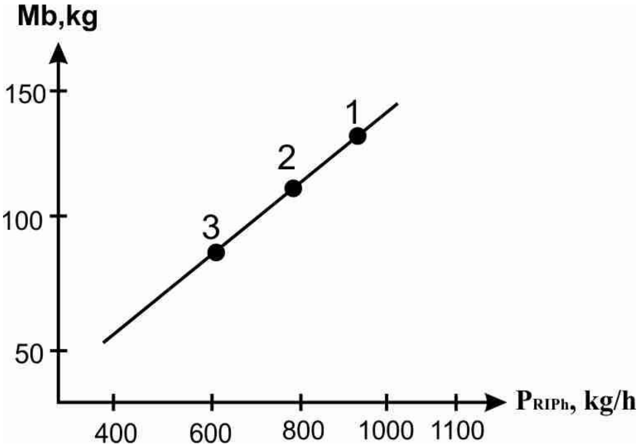

Fig 1 – Experimental dependence of bath mass on the slag productivity of reactor inversion phase
$1-I_{noz}/G_b=(0.26)$; $2-I_{noz}/G_b=(0.42)$; $3-I_{noz}/G_b=(0.44)$

Table 1 - Comparative characteristics of experiments

Experiment numbers, slag type	$\frac{P_{RIPh}, kg/h}{B_{ng}, nm^3/h}$	M_B, kg	I_C/G_B	E, %	τ_{dw}, min	P_a, bar	P_V, $\frac{kg\,Zn}{nm^3 h}$

№ 1, "rich" slag, C_{in}=11,63%	$\frac{1058}{280}$	130	0,26	55	7,37	0,4	14,0
№ 2, "rich" slag, C_{in}=11,63%	$\frac{693}{300}$	112	0,42	74	8,83	0,52	9,14
№ 3, "rich" slag, C_{in}=11,63%	$\frac{612}{300}$	88	0,44	76	7,84	0,42	8,1

Upon calculation the $P_{V,}$ for the unit "reactor inversion phase-rotary kiln", (RIPh-RK)- 5,4 m^3.

Comparison of thermal characteristics of the pilot plant and operating furnaces

Let's choose from the table 1 one of the experimental results, for example No. 2, and compare it with the specific productivity and specific fuel consumption of the Chimkent lead plant's fuming furnace and Leninogorsk Polymetallic JSC's (LP JSC) waelz-kiln :

- fuming furnace of Chimkent lead plant, processing smelt of shaft furnaces, a 75-tonne charge, at 3-hour fumigation cycle. The average hourly output is 25 t / h, the internal volume of the furnace is 147 m3, the temperature of the blast air is 270^{0}C, C^{in}_{Zn}= 10%, zinc extraction degree E = 75%. The specific productivity is 12.75 kg Zn / m3.h, the specific consumption of natural gas is 2300 nm3 / t Zn.
- LP JSC's waelz-kiln, processing cold slag, technological volume of the furnace 816m3 ($L \times D = 70m \times 5m$), average daily slag capacity 750t. The average hourly output is 31.25 tons. The consumption of the coke ~ 480 kg / t slag, oil, mazut ~ 33 kg / t slag. Oxygen enriched blast air, up to 30%. C_{in}^{Zn} = 10%, E = 80%. Specific productivity 3.82 kgZn / m3.h, specific consumption of coke 6070 kg / t Zn.

Let’s compare the fuming and waelz processes:

$$\frac{P_V^f}{P_V^w}=\frac{12{,}75}{3{,}82}=3{,}33.\ \frac{в_{r.f.}^w}{в_{r.f.}^f}=\frac{6070}{2300\cdot 1{,}2}=2{,}2$$

Here: indices of f,w - fuming and waelz processes; r.f. - reference fuel; 1.2 - the ratio coefficient for conversion the natural gas into reference fuel.

From this comparison of practical data, we see that during processing of cold slag, (waelz process), more than 2 times more fuel is expended than when processing molten slag, (fuming process).

According to calculations, unit specific capacity RIPh – RK $P_V^{RIPh\text{-}RK}=9{,}14\frac{kg\ Zn}{nm^3.h}$, and specific consumption of natural gas at RIPh – RK $в_{ng}^{RIPh\text{-}RK}=5028 nm^3/т\ Zn$. Then:

$$\frac{P_V^{RIPh\text{-}RK}}{P_V^w}=\frac{9{,}14}{3{,}82}=2{,}39;\ \frac{в_{r.f.}^{RIPh\text{-}RK}}{в_{r.f.}^w}=\frac{5028\cdot 1{,}2}{6070}=0{,}99$$

From these results of the calculations it can be seen that for a pilot plant with the capacity for cold slag $P_{RIPh}=0{,}693\ t/h$ specific fuel consumption, $в_{r.f.}^{RIPh\text{-}RK}$, not much different from that in waelz-kiln with the capacity of 31.25 t / h, and specific productivity $P_V^{RIPh\text{-}RK}$ more than twice as high as in waelz-kiln.

According to the estimated evaluation carried out in the article «ENERGY-SAVING METHOD FOR TECHNOGENIC WASTE PROCESSING», (see Track 536-538), for the industrial sample of reactor inversion phase with slag capacity 31,25 t/h the specific consumption of natural gas will be approximately 2 times lesser than that in fuming furnace, and the specific fuel consumption is approximately 4 time lower than that in Waelz-kiln

Thus, with the growth of aggregate capacity of the "reactor inversion phase-rotary kiln", there is a tendency to reduce the specific fuel consumption.
